# Compbdt: an R program to compare two binary diagnostic tests subject to a paired design

**DOI:** 10.1186/s12874-020-00988-y

**Published:** 2020-06-05

**Authors:** J. A. Roldán-Nofuentes

**Affiliations:** Department of Statistics (Biostatistics), School of Medicine, University of Granada, Avenida de la Investigación 11, 18016 Granada, Spain

**Keywords:** Binary diagnostic test, Likelihood ratios, Paired design, Predictive values, Sensitivity and specificity

## Abstract

**Background:**

The comparison of the performance of two binary diagnostic tests is an important topic in Clinical Medicine. The most frequent type of sample design to compare two binary diagnostic tests is the paired design. This design consists of applying the two binary diagnostic tests to all of the individuals in a random sample, where the disease status of each individual is known through the application of a gold standard*.* This article presents an R program to compare parameters of two binary tests subject to a paired design.

**Results:**

The “compbdt” program estimates the sensitivity and the specificity, the likelihood ratios and the predictive values of each diagnostic test applying the confidence intervals with the best asymptotic performance. The program compares the sensitivities and specificities of the two diagnostic tests simultaneously, as well as the likelihood ratios and the predictive values, applying the global hypothesis tests with the best performance in terms of type I error and power. When the global hypothesis test is significant, the causes of the significance are investigated solving the individual hypothesis tests and applying the multiple comparison method of Holm. The most optimal confidence intervals are also calculated for the difference or ratio between the respective parameters. Based on the data observed in the sample, the program also estimates the probability of making a type II error if the null hypothesis is not rejected, or estimates the power if the if the alternative hypothesis is accepted. The “compbdt” program provides all the necessary results so that the researcher can easily interpret them. The estimation of the probability of making a type II error allows the researcher to decide about the reliability of the null hypothesis when this hypothesis is not rejected. The “compbdt” program has been applied to a real example on the diagnosis of coronary artery disease.

**Conclusions:**

The “compbdt” program is one which is easy to use and allows the researcher to compare the most important parameters of two binary tests subject to a paired design. The “compbdt” program is available as supplementary material.

## Background

A diagnostic test is a medical test that is applied to an individual in order to determine the presence or absence of a disease. When the result of a diagnostic test is positive or negative, the diagnostic test is called a binary diagnostic test. A stress test for the diagnosis of coronary disease is an example of binary diagnostic test. The performance of a binary diagnostic test is measured in terms of two fundamental parameters: sensitivity and specificity. The sensitivity (*Se*) is the probability of the diagnostic test being positive when the individual has the disease, and the specificity (*Sp*) is the probability of the diagnostic test being negative when the individual does not have it. The *Se* and the *Sp* of a diagnostic test are estimated in relation to a gold standard, which is a medical test which objectively determines whether or not an individual has the disease or not. An angiography for coronary disease is an example of a gold standard. Other parameters that are used to assess the performance of a diagnostic test are the likelihood ratios (*LRs*) and the predictive values (*PVs*) [1, 2]. When the diagnostic test is positive, the likelihood ratio, called the positive likelihood ratio (*PLR*), is the ratio between the probability of correctly classifying an individual with the disease and the probability of incorrectly classifying an individual who does not have it, i.e. *PLR* = *Se*/(1 − *Sp*). When the diagnostic test is negative, the likelihood ratio, called the negative likelihood ratio (*NLR*), is the ratio between the probability of incorrectly classifying an individual who has the disease and the probability of correctly classifying an individual who does not have it, i.e. *NLR* = (1 − *Se*)/*Sp*. The *LRs* only depend on *Se* and *Sp* of the diagnostic test and they are equivalent to a relative risk. The positive predictive value (*PPV*) is the probability of an individual having the disease when the result of the diagnostic test is positive, and the negative predictive value (*NPV*) is the probability of an individual not having the disease when the result of the diagnostic test is negative. The *PVs* represent the accuracy of the diagnostic test when it is applied to a cohort of individuals, and they are measures of the clinical accuracy of the diagnostic test. The *PVs* depend on the *Se* and the *Sp* of the diagnostic test and on the disease prevalence (*p*), and are easily calculated applying Bayes’ Theorem i.e.

$$ PPV=\frac{p\times Se}{p\times Se+\left(1-p\right)\times \left(1- Sp\right)}\mathrm{and} NPV=\frac{\left(1-p\right)\times Sp}{p\times \left(1- Se\right)+\left(1-p\right)\times Sp}. $$

Whereas the *Se* and the *Sp* quantify how well the diagnostic test reflects the true disease status (present or absent), the *PVs* quantify the clinical value of the diagnostic test, since both the individual and the clinician are more interested in knowing how probable it is to have the disease given a diagnostic test result.

The comparison of the performance of two diagnostic tests with respect to a gold standard is an important topic in Clinical Medicine and Epidemiology. The most frequent type of sample design to compare two diagnostic test*s* with respect to a gold standard is paired design [1, 2]. This design consists of applying the two diagnostic tests, *Test* 1 and *Test* 2, to all of the individuals in a random sample sized *n*, where the disease status of each individual is known through the application of a gold standard. Therefore, subject to a paired design the two diagnostic tests and the gold standard are applied to all of the individuals in a single random sample, whose size (*n*) has been set by the researcher. Paired design is the most efficient type of design to compare two binary diagnostic tests as it minimizes the impact of the between-individual variability, therefore this manuscript focuses on paired design. The comparison of two diagnostic tests subject to this type of design leads to the frequencies that are shown in Table 1, where *s*_*ij*_ (*r*_*ij*_) be the number of diseased (non-diseased) patients in which the *Test* 1 gives a result *i* (1 positive and 0 negative) and *Test* 2 gives a result *j* (1 positive and 0 negative).

**Table 1:** Frequencies subject to a paired design

	Test 1 positive		Test 1 negative		
	Test 2 positive	Test 2 negative	Test 2 positive	Test 2 negative	Total
Disease	*s*_11_	*s*_10_	*s*_01_	*s*_00_	*s*
No disease	*r*_11_	*r*_10_	*r*_01_	*r*_00_	*r*
Total	*n*_11_	*n*_10_	*n*_01_	*n*_00_	*n*

This article presents a program called “compbdt” (Comparison of two Binary Diagnostic Tests) written in R [3] which allows us to estimate and compare the performance (measured in terms of the previous parameters) of two diagnostic test*s* subject to a paired design applying the statistical methods with the best asymptotic performance, i.e. for the confidence intervals we used the intervals that have a better coverage and average width, and for the hypothesis tests we used the methods that have the best behaviour in terms of type I error and power. In the next section, the methods of estimation and of comparison of the parameters are summarized, and the “compbdt” program is explained. The results are applied to a real example of the diagnosis of coronary artery disease, and finally some conclusions are given.

## Implementation

The estimation and comparison of parameters of two diagnostic tests has been the subject of numerous studies in Statistics literature. We will now describe the statistical methods implemented in the “compbdt” program to estimate the parameters and to compare the respective parameters subject to a paired design. The methods used are those that have a better asymptotic behaviour in terms of coverage for the confidence intervals and in terms of type I error and power for hypothesis tests.

### Estimation of the parameters

The estimation of the sensitivity, the specificity and the predictive values of each diagnostic test consists of the estimation of a binomial proportion. There are numerous confidence intervals proposed to estimate a binomial proportion. Yu et al. [4] proposed a new interval, based on a modification of the Wilson interval, to estimate a binomial proportion, demonstrating that this interval shows a better asymptotic performance than the rest of the existing intervals. For the sensitivity of each diagnostic test, the estimators are.

$$ \hat{S}{e}_1=\frac{s_{11}+{s}_{10}}{s}\kern0.5em \mathrm{and}\kern0.5em \hat{S}{e}_2=\frac{s_{11}+{s}_{01}}{s},\kern0.5em $$

and their standard errors (*SE*) are

$$ SE\left(\hat{S}{e}_i\right)=\sqrt{\frac{\hat{S}{e}_i\left(1-\hat{S}{e}_i\right)}{n\hat{p}}}, $$

with *i* = 1, 2, and where $\hat{p}=s/n$ is the estimator of the disease prevalence. The Yu et al. confidence interval for sensitivity *Se*_*i*_, with *i* = 1, 2, is

$$ {Se}_i\in 0.5+\frac{s+{z}_{1-\alpha /2}^4/53}{s+{z}_{1-\alpha /2}^4}\left(\hat{S}{e}_i-0.5\right)\pm \frac{z_{1-\alpha /2}}{s+{z}_{1-\alpha /2}^2}\sqrt{s\left(1-\hat{S}{e}_i\right)\hat{S}{e}_i+\frac{z_{1-\alpha /2}^2}{4}}, $$

where *z*_1 − *α*/2_ is the 100(1 − *α*/2)*th* percentile of the standard normal distribution. For the specificities, the estimators are

$$ \hat{S}{p}_1=\frac{r_{01}+{r}_{00}}{r}\kern0.5em \mathrm{and}\kern0.5em \hat{S}{p}_2=\frac{r_{10}+{r}_{00}}{r}, $$

and their standard errors (*SE*) are

$$ SE\left(\hat{S}{p}_i\right)=\sqrt{\frac{\hat{S}{p}_i\left(1-\hat{S}{p}_i\right)}{n\left(1-\hat{p}\right)}}. $$

The intervals for the specificities are obtained analogously by replacing $\hat{S}{e}_i$ with $\hat{S}{p}_i$ and *s* with *r*.

For the predictive values, the estimators of the *PPVs* are

$$ \hat{P}{PV}_1=\frac{s_{10}+{s}_{11}}{s_{10}+{s}_{11}+{r}_{10}+{r}_{11}}\kern0.5em \mathrm{and}\kern0.5em \hat{P}{PV}_2=\frac{s_{01}+{s}_{11}}{s_{01}+{s}_{11}+{r}_{01}+{r}_{11}}, $$

and their standard errors are.

$$ SE\left(\hat{P}{PV}_1\right)=\sqrt{\frac{\left({s}_{10}+{s}_{11}\right)\left({r}_{10}+{r}_{11}\right)}{n{\left({s}_{10}+{s}_{11}+{r}_{10}+{r}_{11}\right)}^3}}\kern0.5em \mathrm{and} SE\left(\hat{P}{PV}_2\right)=\sqrt{\frac{\left({s}_{01}+{s}_{11}\right)\left({r}_{01}+{r}_{11}\right)}{n{\left({s}_{01}+{s}_{11}+{r}_{10}+{r}_{11}\right)}^3}}\kern0.5em . $$

The estimators of the *NPVs* are

$$ \hat{N}{PV}_1=\frac{r_{00}+{r}_{01}}{s_{00}+{s}_{01}+{r}_{00}+{r}_{01}}\kern0.5em \mathrm{and}\kern0.5em \hat{N}{PV}_2=\frac{r_{00}+{r}_{10}}{s_{00}+{s}_{10}+{r}_{00}+{r}_{10}}, $$

and their standard errors are.

$$ SE\left(\hat{N}{PV}_1\right)=\sqrt{\frac{\left({s}_{00}+{s}_{01}\right)\left({r}_{00}+{r}_{01}\right)}{n{\left({s}_{00}+{s}_{01}+{r}_{00}+{r}_{01}\right)}^3}}\kern0.5em \mathrm{and}\kern0.5em SE\left(\hat{N}{PV}_2\right)=\sqrt{\frac{\left({s}_{00}+{s}_{10}\right)\left({r}_{00}+{r}_{10}\right)}{n{\left({s}_{00}+{s}_{10}+{r}_{00}+{r}_{10}\right)}^3}}. $$

For *PPV* and *NPV* of *Test* 1, the Yu et al. confidence intervals are

$$ 0.5+\frac{n_{1\cdotp }+{z}_{1-\alpha /2}^4/53}{n_{1\cdotp }+{z}_{1-\alpha /2}^4}\left(\hat{P}{PV}_1-0.5\right)\pm \frac{z_{1-\alpha /2}}{n_{1\cdotp }+{z}_{1-\alpha /2}^2}\sqrt{n_{1\cdotp}\left(1-\hat{P}{PV}_1\right)\hat{P}{PV}_1+\frac{z_{1-\alpha /2}^2}{4}} $$

and

$$ 0.5+\frac{n_{0\cdotp }+{z}_{1-\alpha /2}^4/53}{n_{0\cdotp }+{z}_{1-\alpha /2}^4}\left(\hat{N}{PV}_1-0.5\right)\pm \frac{z_{1-\alpha /2}}{n_{0\cdotp }+{z}_{1-\alpha /2}^2}\sqrt{n_{0\cdotp}\left(1-\hat{N}{PV}_1\right)\hat{N}{PV}_1+\frac{z_{1-\alpha /2}^2}{4}} $$

where *n*_1·_ = (*n*_11_ + *n*_10_) and *n*_0·_ = *n*_01_ + *n*_00_, respectively. The confidence intervals for *PPV* and *NPV* of *Test* 2 are obtained analogously by replacing *n*_1·_ with *n*_·1_ = *n*_11_ + *n*__{01}_ and $\hat{P}{PV}_1$ with $\hat{P}{PV}_2$, and replacing *n*_0·_ with *n*_·0_ = *n*_10_ + *n*_00_ and $\hat{N}{PV}_1$ with $\hat{N}{PV}_2$, respectively.

Regarding the likelihood ratios, the estimators of *PLRs* are

$$ \hat{P}{LR}_1=\frac{r\left({s}_{11}+{s}_{10}\right)}{s\left({r}_{11}+{r}_{10}\right)}\kern0.5em \mathrm{and}\kern0.5em \hat{P}{LR}_2=\frac{r\left({s}_{11}+{s}_{01}\right)}{s\left({r}_{11}+{r}_{01}\right)}, $$

and their standard errors are.

$$ SE\left(\hat{P}{LR}_i\right)=\sqrt{\frac{\hat{S}{e}_i^2\times \hat{V} ar\left(\hat{S}{p}_i\right)+{\left(1-\hat{S}{p}_i\right)}^2\times \hat{V} ar\left(\hat{S}{e}_i\right)}{{\left(1-\hat{S}{p}_i\right)}^4}},\kern0.5em i=1,2, $$

where $\hat{V} ar\left(\hat{S}{e}_i\right)={\left[ SE\left(\hat{S}{e}_i\right)\right]}^2$ and $\hat{V} ar\left(\hat{S}{p}_i\right)={\left[ SE\left(\hat{S}{p}_i\right)\right]}^2$. The estimators of *NLRs* are.

$$ \hat{N}{LR}_1=\frac{r\left({s}_{01}+{s}_{00}\right)}{s\left({r}_{01}+{r}_{00}\right)}\kern0.5em \mathrm{and}\kern0.5em \hat{N}{LR}_2=\frac{r\left({s}_{10}+{s}_{00}\right)}{s\left({r}_{10}+{r}_{00}\right)}, $$

and their standard errors are

$$ SE\left(\hat{N}{LR}_i\right)=\sqrt{\frac{{\left(1-\hat{S}{e}_i\right)}^2\times \hat{V} ar\left(\hat{S}{p}_i\right)+\hat{S}{p}_i^2\times \hat{V} ar\left(\hat{S}{e}_i\right)}{\hat{S}{p}_i^4}},\kern0.5em i=1,2. $$

The *LRs* are the ratio of two independent binomial proportions, i.e. a relative risk. Martín-Andrés and Álvarez-Hernández [5] compared 73 confidence intervals for the ratio of two independent binomial proportions, and concluded that the interval with the best performance is the interval based on an approximation to the score method adding 0.5 to the observed frequencies. For *Test* 1, these confidence intervals are:

$$ {\displaystyle \begin{array}{c}{PLR}_1\in \\ {}\frac{{\tilde{n}\tilde{s}}_{1\cdotp }{\tilde{r}}_{1\cdotp }+\frac{z_{1-\alpha /2}^2}{2}\left({\tilde{s}\tilde{s}}_{1\cdotp }+{{\tilde{r}\tilde{r}}_{1\cdotp}}^{\prime }-2{\tilde{s}}_{1\cdotp }{\tilde{r}}_{1\cdotp}\right)\pm {z}_{1-\alpha /2}\sqrt{{\tilde{n}}^2{\tilde{s}}_{1\cdotp }{\tilde{r}}_{1\cdotp}\left[{\tilde{s}}_{1\cdotp }+{\tilde{r}}_{1\cdotp }-\tilde{n}\tilde{S}{e}_1\left(1-\tilde{S}{p}_1\right)\right]+\frac{z_{1-\alpha /2}^2}{4}{\left({\tilde{s}\tilde{s}}_{1\cdotp }-{\tilde{r}\tilde{r}}_{1\cdotp}\right)}^2}}{{\tilde{r}}_{1\cdotp}\left[\tilde{n}\tilde{s}\left(1-\tilde{S}{p}_1\right)-{z}_{1-\alpha /2}^2\left(\tilde{s}-{\tilde{r}}_{1\cdotp}\right)\right]}\end{array}} $$

and

$$ {\displaystyle \begin{array}{c}{NLR}_1\in \\ {}\frac{{\tilde{n}\tilde{s}}_{0\cdotp }{\tilde{r}}_{0\cdotp }+\frac{z_{1-\alpha /2}^2}{2}\left({\tilde{s}\tilde{s}}_{0\cdotp }+{\tilde{r}\tilde{r}}_{0\cdotp }-2{\tilde{s}}_{0\cdotp }{\tilde{r}}_{0\cdotp}\right)\pm {z}_{1-\alpha /2}\sqrt{{\tilde{n}}^2{\tilde{s}}_{0\cdotp }{\tilde{r}}_{0\cdotp}\left[{\tilde{s}}_{0\cdotp }+{\tilde{r}}_{0\cdotp }-\tilde{n}\left(1-\tilde{S}{e}_1\right)\tilde{S}{p}_1\right]+\frac{z_{1-\alpha /2}^2}{4}{\left({\tilde{s}\tilde{s}}_{0\cdotp }-{\tilde{r}\tilde{r}}_{0\cdotp}\right)}^2}}{{\tilde{r}}_{0\cdotp}\left[\tilde{n}\tilde{s}\tilde{S}{p}_1-{z}_{1-\alpha /2}^2\left(\tilde{s}-{\tilde{r}}_{0\cdotp}\right)\right]}\end{array}} $$

where ${\tilde{s}}_{1\cdotp }={s}_{1\cdotp }+0.5$, ${\tilde{s}}_{0\cdotp }={s}_{0\cdotp }+0.5$, ${\tilde{r}}_{0\cdotp }={r}_{0\cdotp }+0.5$, ${\tilde{r}}_{1\cdotp }={r}_{1\cdotp }+0.5$, $\tilde{s}=s+1$, $\tilde{r}=r+1$, $\tilde{n}=n+2$, $\tilde{S}{e}_1={\tilde{s}}_{1\cdotp }/\tilde{s}$ and $\tilde{S}{p}_1={\tilde{r}}_{0\cdotp }/\tilde{r}$. If the lower limit of the interval for *PLR*_1_ is less than ${\tilde{s}}_{1\cdotp }/\left(\tilde{n}-{\tilde{r}}_{1\cdotp}\right)$ or greater than $\hat{P}{LR}_1$, then the lower limit of the confidence interval is

$$ \frac{{\tilde{s}}_{1\cdotp}\left(1-\tilde{S}{p}_1\right)+\frac{z_{1-\alpha /2}^2}{2}-{z}_{1-\alpha /2}\sqrt{\frac{z_{1-\alpha /2}^2}{4}+{\tilde{s}}_{1\cdotp}\left(1-\tilde{S}{p}_1-\tilde{S}{e}_2\right)}}{\tilde{s}{\left(1-\tilde{S}{p}_1\right)}^2+{z}_{1-\alpha /2}^2}, $$

and if the upper limit of this interval is greater than $\left(\tilde{n}-{\tilde{s}}_{1\cdotp}\right)/{\tilde{r}}_{1\cdotp }$ or lower than $\hat{P}{LR}_1$, then the upper limit of the confidence interval is

$$ \frac{{\tilde{r}}_{1\cdotp}\tilde{S}{e}_1+\frac{z_{1-\alpha /2}^2}{2}+{z}_{1-\alpha /2}\sqrt{\frac{z_{1-\alpha /2}^2}{4}+{\tilde{r}}_{1\cdotp}\left(\tilde{S}{e}_1+\tilde{S}{p}_1-1\right)}}{\tilde{r}{\left(1-\tilde{S}{p}_1\right)}^2}. $$

Regarding the confidence interval for *NLR*_1_, if the lower limit of this interval is less than ${\tilde{s}}_{0\cdotp }/\left(\tilde{n}-{\tilde{r}}_{0\cdotp}\right)$ or greater than $\hat{N}{LR}_1$, then the lower limit of the confidence interval is

$$ \frac{{\tilde{s}}_{0\cdotp}\tilde{S}{p}_1+\frac{z_{1-\alpha /2}^2}{2}-{z}_{1-\alpha /2}\sqrt{\frac{z_{1-\alpha /2}^2}{4}+{\tilde{s}}_{0\cdotp}\left(\tilde{S}{p}_1+\tilde{S}{e}_1-1\right)}}{\tilde{s}\tilde{S}{p}_1^2+{z}_{1-\alpha /2}^2}, $$

and if the upper limit of this interval is greater than $\left(\tilde{n}-{\tilde{s}}_{0\cdotp}\right)/{\tilde{r}}_{0\cdotp }$ or less than $\hat{N}{LR}_1$, then the upper limit of the confidence interval is

$$ \frac{{\tilde{r}}_{0\cdotp}\left(1-\tilde{S}{e}_1\right)+\frac{z_{1-\alpha /2}^2}{2}+{z}_{1-\alpha /2}\sqrt{\frac{z_{1-\alpha /2}^2}{4}+{\tilde{r}}_{0\cdotp}\left(1-\tilde{S}{e}_1-\tilde{S}{p}_1\right)}}{\tilde{r}\tilde{S}{p}_1^2}. $$

The confidence intervals for *LRs* of *Test* 2 are obtained analogously by replacing ${\tilde{s}}_{1\cdotp }$ with ${\tilde{s}}_{\cdotp 1}={s}_{\cdotp 1}+0.5$, ${\tilde{r}}_{1\cdotp }$ with ${\tilde{r}}_{\cdotp 1}={r}_{\cdotp 1}+0.5$, ${\tilde{s}}_{0\cdotp }$ with ${\tilde{s}}_{\cdotp 0}={s}_{\cdotp 0}+0.5$, ${\tilde{r}}_{0\cdotp }$ with ${\tilde{r}}_{\cdotp 0}={r}_{\cdotp 0}+0.5$, $\tilde{S}{e}_1$ with $\tilde{S}{e}_2={\tilde{s}}_{\cdotp 1}/\tilde{s}$ and $\tilde{S}{p}_1$ with $\tilde{S}{p}_2={\tilde{r}}_{\cdotp 0}/\tilde{r}$.

The “compbdt” program also estimates the prevalence of the disease. The estimator of the prevalence is $\hat{p}=s/n$, the standard error is $\sqrt{\hat{p}\left(1-\hat{p}\right)/n}$ and the Yu et al. confidence interval for the prevalence is

$$ p\in 0.5+\frac{n+{z}_{1-\alpha /2}^4/53}{n+{z}_{1-\alpha /2}^4}\left(\hat{p}-0.5\right)\pm \frac{z_{1-\alpha /2}}{n+{z}_{1-\alpha /2}^2}\sqrt{n\left(1-\hat{p}\right)\hat{p}+\frac{z_{1-\alpha /2}^2}{4}}. $$

### Comparison of the parameters

The comparison of parameters of two diagnostic tests subject to a paired design has been the subject of different studies. The hypothesis tests with the best performance, in terms of type I and power error, to compare the parameters of two diagnostic tests are presented below.

#### Comparison of the sensitivities and the specificities

Traditionally, the comparison of two sensitivities and of two specificities was carried out solving the hypothesis tests *H*_0_ : *Se*_1_ = *Se*_2_ vs *H*_1_ : *Se*_1_ ≠ *Se*_2_ and *H*_0_ : *Sp*_1_ = *Sp*_2_ vs *H*_1_ : *Sp*_1_ ≠ *Sp*_2_ each one of them to an *α* error, applying a comparison test of two paired binomial proportions (e.g. the McNemar test) [2]. Recently, Roldán-Nofuentes and Sidaty-Regad [6] have studied different methods to compare the two sensitivities and the two specificities individually and also simultaneously, and carried out simulation experiments to compare these methods. The results of the simulation experiments showed that disease prevalence and sample size have an important effect on the type I errors and powers of the methods analysed, and from the results obtained some general rules of application were given in terms of the prevalence and the sample size. These rules are:

a). When the prevalence is small (≤10%) and the sample size *n* is ≤100, solve the tests *H*_0_ : *Se*_1_ = *Se*_2_ and *H*_0_ : *Sp*_1_ = *Sp*_2_ individually applying the Wald test (or the likelihood ratio test) along with the Bonferroni or Holm method [7] to an *α* error. However, the second method has the disadvantage that it can only be applied if the frequencies of the discordant pairs are greater than zero. For *H*_0_ : *Se*_1_ = *Se*_2_ the Wald test statistic is

$$ {\chi}_{WSe}^2=\frac{s{\left({s}_{10}-{s}_{01}\right)}^2}{4{s}_{10}{s}_{01}+\left({s}_{11}+{s}_{00}\right)\left({s}_{10}+{s}_{01}\right)}, $$

and for *H*_0_ : *Sp*_1_ = *Sp*_2_ the Wald test statistic is

$$ {\chi}_{WSp}^2=\frac{r{\left({r}_{10}-{r}_{01}\right)}^2}{4{r}_{10}{r}_{01}+\left({r}_{11}+{r}_{00}\right)\left({r}_{10}+{r}_{01}\right)}. $$

Likelihood ratio test statistics are

$$ {\chi}_{LRTSe}^2=2\left[{s}_{10}\ln \left(\frac{2{s}_{10}}{s_{10}+{s}_{01}}\right)+{s}_{01}\ln \left(\frac{2{s}_{01}}{s_{10}+{s}_{01}}\right)\right] $$

and

$$ {\chi}_{LRTSp}^2=2\left[{r}_{10}\ln \left(\frac{2{r}_{10}}{r_{10}+{r}_{01}}\right)+{r}_{01}\ln \left(\frac{2{r}_{01}}{r_{10}+{r}_{01}}\right)\right], $$

respectively. These statistics have a standard normal distribution. Both methods, the Wald test and the likelihood ratio test, have a very similar asymptotic performance. However, the second method has the disadvantage that it can only be applied if the frequencies of the discordant pairs are greater than zero.

b) In any other situation, solve the global test *H*_0_ : (*Se*_1_ = *Se*_2_ and *Sp*_1_ = *Sp*_2_) vs *H*_1_ : (*Se*_1_ ≠ *Se*_2_ and/or *Sp*_1_ ≠ *Sp*_2_) to an *α* error applying the Wald test or the likelihood ratio test, i.e.

$$ {\chi}_W^2=\frac{s{\left({s}_{10}-{s}_{01}\right)}^2}{4{s}_{10}{s}_{01}+\left({s}_{11}+{s}_{00}\right)\left({s}_{10}+{s}_{01}\right)}+\frac{r{\left({r}_{10}-{r}_{01}\right)}^2}{4{r}_{10}{r}_{01}+\left({r}_{11}+{r}_{00}\right)\left({r}_{10}+{r}_{01}\right)} $$

and

$$ {\chi}_{LRT}^2=2\left[{s}_{10}\ln \left(\frac{2{s}_{10}}{s_{10}+{s}_{01}}\right)+{s}_{01}\ln \left(\frac{2{s}_{01}}{s_{10}+{s}_{01}}\right)+{r}_{10}\ln \left(\frac{2{r}_{10}}{r_{10}+{r}_{01}}\right)+{r}_{01}\ln \left(\frac{2{r}_{01}}{r_{10}+{r}_{01}}\right)\right]. $$

The distribution of both statistics is a chi-square with two degrees of freedom when the null hypothesis is true. In this situation, if the global test is not significant then the equality of the accuracies of both diagnostic tests is not rejected, and if the global test is significant then the causes of the significance will be investigated: 1) testing the tests *H*_0_ : *Se*_1_ = *Se*_2_ and *H*_0_ : *Sp*_1_ = *Sp*_2_ individually applying the Wald test (or the likelihood ratio test) along with the Holm method [7] (or Bonferroni) to an *α* error if the sample size is ≤100 or if the sample size is ≥1000; or 2) testing the tests *H*_0_ : *Se*_1_ = *Se*_2_ and *H*_0_ : *Sp*_1_ = *Sp*_2_ individually applying the McNemar test with continuity correction (*cc*) to an *α* error if 100 < *n* < 1000. McNemar test statistics with *cc* are

$$ {\chi}_{MccSe}^2=\frac{{\left(\left|{s}_{10}-{s}_{01}\right|-1\right)}^2}{s_{10}+{s}_{01}}\kern0.5em \mathrm{and}\kern0.5em {\chi}_{MccSp}^2=\frac{{\left(\left|{r}_{10}-{r}_{01}\right|-1\right)}^2}{r_{10}+{r}_{01}}, $$

respectively. In all of these test statistics we consider the frequencies of discordant pairs *s*_*ij*_ and *r*_*ij*_ with *i* ≠ *j*, which are the base of the development of the McNemar test.

Regarding the confidence intervals for the difference between the two sensitivities (specificities), these consist of intervals for the difference between the two paired binomial proportions. Fagerland et al. [8] compared different intervals and recommended using the Wald interval with Bonett-Laplace adjustment. For the difference between the two sensitivities, the Wald interval with Bonett-Laplace adjustment is

$$ {Se}_1-{Se}_2\in \frac{s_{10}-{s}_{01}}{s+2}\pm {z}_{1-\alpha /2}\sqrt{\frac{s_{10}+{s}_{01}+2}{{\left(s+2\right)}^2}-\frac{{\left({s}_{10}-{s}_{01}\right)}^2}{{\left(s+2\right)}^3}}, $$

and for the difference between the two specificities the confidence interval is

$$ {Sp}_1-{Sp}_2\in \frac{r_{10}-{r}_{01}}{r+2}\pm {z}_{1-\alpha /2}\sqrt{\frac{r_{10}+{r}_{01}+2}{{\left(r+2\right)}^2}-\frac{{\left({r}_{10}-{r}_{01}\right)}^2}{{\left(r+2\right)}^3}}. $$

These intervals are included in the interval [− 1, 1].

The “compbdt” program uses the method of Roldán-Nofuentes and Sidaty-Regad [6] and the confidence interval of Wald interval with Bonett-Laplace adjustment for the difference between the two sensitivities (specificities).

#### Comparison of the likelihood ratios

The comparison of the *LRs* of two diagnostic tests subject to a paired design has been the subject of several studies. Leisenring and Pepe [8] have studied the estimation of the *LRs* of a diagnostic test using a regression model, and Pepe [1] has adapted this model to compare the *LRs* individually of two binary diagnostic tests, i.e. to solve the tests *H*_0_ : *PLR*_1_ = *PLR*_2_ vs *H*_1_ : *PLR*_1_ ≠ *PLR*_2_ and *H*_0_ : *NLR*_1_ = *NLR*_2_ vs *H*_1_ : *NLR*_1_ ≠ *NLR*_2_. Roldán-Nofuentes and Luna [9] have compared the *LRs* individually, and also simultaneously, i.e. solving the global hypothesis test *H*_0_ : (*PLR*_1_ = *PLR*_2_ and *NLR*_1_ = *NLR*_2_) vs *H*_1_ : (*PLR*_1_ ≠ *PLR*_2_ and/or *NLR*_1_ ≠ *NLR*_2_), applying the maximum likelihood method. Dolgun et al. [10] have extended the method of Leisenring and Pepe to compare the *LRs* simultaneously. The test statistics of the individual hypotheses tests of Pepe and the test statistics of the individual hypotheses tests of Roldán-Nofuentes and Luna have a very similar asymptotic behaviour. The test statistic of the global hypothesis test of Dolgun et al. and the test statistic of the global hypothesis test of Roldán-Nofuentes and Luna have a very similar asymptotic behaviour. Therefore, the “compbdt” uses the tests proposed by Roldán-Nofuentes and Luna.

The method of Roldán-Nofuentes and Luna [9] compares the *LRs* considering the Napierian logarithm of the ratios of the *PLRs* and of the *NLRs*. The test statistic for the global hypothesis test of simultaneous comparison of the *LRs* is obtained applying the Wald test, i.e.

$$ {\chi}_W^2={\hat{\boldsymbol{\upomega}}}^T{\hat{\Sigma}}_{\hat{\boldsymbol{\upomega}}}^{-1}\hat{\boldsymbol{\upomega}}, $$

and whose distribution is a chi-square with two degrees of freedom when the null hypothesis is true, where $\hat{\boldsymbol{\upomega}}={\left(\ln \left(\hat{P}{LR}_1/\hat{P}{LR}_2\right),\ln \left(\hat{N}{LR}_1/\hat{N}{LR}_2\right)\right)}^T$ and ${\hat{\Sigma}}_{\hat{\boldsymbol{\upomega}}}$ it is the estimated variance-covariance matrix obtained by applying the delta method. Roldán-Nofuentes and Amro [11] proposed the following procedure to compare the *LRs*: 1) Solve the global hypothesis test to an *α* error calculating the Wald test statistic; 2) If the global hypothesis test is not significant to an *α* error, then the homogeneity of the *LRs* of the two diagnostic tests is not rejected, but if the global hypothesis test is significant to an *α* error, then the study of the causes of the significance is performed by solving the two individual hypothesis tests along with a multiple comparison method (e.g. Holm method [7]) to an *α* error. In this situation, the test statistic for comparing the two *PLRs* is

$$ \frac{\ln \left(\hat{P}{LR}_1/\hat{P}{LR}_2\right)}{\sqrt{\hat{V} ar\left[\ln \left(\hat{P}{LR}_1/\hat{P}{LR}_2\right)\right]}} $$

and the test statistic for comparing the two *NLRs* is

$$ \frac{\ln \left(\hat{N}{LR}_1/\hat{N}{LR}_2\right)}{\sqrt{\hat{V}ar\left[\ln \left(\hat{N}{LR}_1/\hat{N}{LR}_2\right)\right]}}. $$

These test statistics are distributed asymptotically according to a standard normal distribution.

Regarding the confidence intervals, Roldán-Nofuentes and Sidaty-Regad [12] studied the comparison of the *LRs* through confidence intervals. For the *PLRs*, it is recommended to use an interval based on the Napierian logarithm of the ratio between both, and for the *NLRs* it is recommended to use a Wald type interval for the ratio between both, i.e.

$$ \frac{PLR_1}{PLR_2}\in \frac{\hat{P}{LR}_1}{\hat{P}{LR}_2}\times \exp \left[\pm {z}_{1-\alpha /2}\sqrt{\hat{V} ar\left(\hat{P}{LR}_1/\hat{P}{LR}_2\right)}\right] $$

and

$$ \frac{NLR_1}{NLR_2}\in \frac{\hat{N}{LR}_1}{\hat{N}{LR}_2}\times \left[1\pm {z}_{1-\alpha /2}\sqrt{\hat{V} ar\left(\hat{N}{LR}_1/\hat{N}{LR}_2\right)}\right], $$

where the variances are calculated by applying the delta method.

#### Comparison of the predictive values

Comparison of the *PVs* has also been the subject of different studies. Leisenring et al. [13], Wang et al. [14], Kosinski [15] and Tsou [16] studied asymptotic methods to compare the *PPVs* and the *NPVs* of two diagnostic tests independently, i.e. solving the two hypothesis tests *H*_0_ : *PPV*_1_ = *PPV*_2_ and *H*_0_ : *NPV*_1_ = *NPV*_2_ each one of them to an *α* error. Takahashi and Yamamoto [17] proposed an exact test to solve this same problem. The Kosinski method has a better asymptotic performance (in terms of type I error and power) than the methods of Leisenring et al. and of Wang et al. The method of Tsou leads to the same results as the Kosinski method. The method of Takahashi and Yamamoto is very conservative (as it is an exact test), even more so than the Kosinski method with small samples. The test statistics of the Kosinski method for *H*_0_ : *PPV*_1_ = *PPV*_2_ is

$$ {T}_{VPP}^{WGS}=\frac{{\left(\hat{P}{PV}_1-\hat{P}{PV}_2\right)}^2}{\left\{\hat{P}{PV}_p\left(1-\hat{P}{PV}_p\right)-2{C}_p^{PPV}\right\}\left(\frac{1}{n_{10}+{n}_{11}}+\frac{1}{n_{01}+{n}_{11}}\right)} $$

and the test statistics for *H*_0_ : *NPV*_1_ = *NPV*_2_ is

$$ {T}_{VPN}^{WGS}=\frac{{\left(\hat{N}{PV}_1-\hat{N}{PV}_2\right)}^2}{\left\{\hat{N}{PV}_p\left(1-\hat{N}{PV}_p\right)-2{C}_p^{NPV}\right\}\left(\frac{1}{n_{00}+{n}_{01}}+\frac{1}{n_{00}+{n}_{10}}\right)}, $$

and where

$$ \hat{P}{PV}_p=\frac{2{s}_{11}+{s}_{10}+{s}_{01}}{2{n}_{11}+{n}_{10}+{n}_{01}},\kern0.5em \hat{N}{PV}_p=\frac{2{r}_{00}+{r}_{01}+{r}_{10}}{2{n}_{00}+{n}_{01}+{n}_{10}}, $$

$$ {C}_p^{PPV}=\frac{s_{11}{\left(1-\hat{P}{PV}_p\right)}^2+{r}_{11}\hat{P}{PV}_p^2}{2{n}_{11}+{n}_{10}+{n}_{01}}\kern0.5em \mathrm{and}\kern0.5em {C}_p^{NPV}=\frac{s_{00}\hat{N}{PV}_p^2+{r}_{00}\left(1-\hat{N}{PV}_p^2\right)}{2{n}_{00}+{n}_{01}+{n}_{10}}. $$

Each statistic is distributed according to a chi-square distribution with one degree of freedom when the corresponding null hypothesis is true.

Roldán-Nofuentes et al. [18] demonstrated that the comparison of the *PVs* of two diagnostic tests subject to a paired design should be carried out simultaneously, i.e. solving the hypothesis test

$$ {H}_0:\left({PPV}_1={PPV}_2\kern0.3em \mathrm{and}\kern0.3em {NPV}_1={NPV}_2\right) vs{H}_1:\left({PPV}_1\ne {PPV}_2\kern0.5em \mathrm{and}/\mathrm{or}\kern0.3em {NPV}_1\ne {NPV}_2\right). $$

Roldán-Nofuentes et al. deduced a statistic applying the Wald test, whose distribution is a chi-square with two degrees of freedom when the null hypothesis is true. This test statistic is

$$ {\chi}_W^2={\hat{\boldsymbol{\upeta}}}^T{\boldsymbol{\upvarphi}}^T{\left(\boldsymbol{\upvarphi} \hat{\sum}{\boldsymbol{\upvarphi}}^T\right)}^{-1}\boldsymbol{\upvarphi} \hat{\boldsymbol{\upeta}}, $$

where $\hat{\boldsymbol{\upeta}}={\left(\hat{P}{PV}_1,\hat{P}{PV}_2,\hat{N}{PV}_1,\hat{N}{PV}_2\right)}^T$, $\hat{\sum}$ is the estimated variance-covariance matrix of $\hat{\boldsymbol{\upeta}}$ calculated by applying the delta method and **φ** is the design matrix, i.e.

$$ \boldsymbol{\upvarphi} =\left(\begin{array}{cccc}1& -1& 0& 0\\ {}0& 0& 1& -1\end{array}\right). $$

The test statistic ${\chi}_W^2$ is distributed asymptotically according to a central chi-square distribution with two degrees of freedom if *H*_0_ is true. Setting an *α* error, if the global test is not significant then we do not reject the equality of the *PVs* of both diagnostic tests; if the global test is significant, then the investigation of the causes of the significance is carried out applying an individual test along with a multiple comparison method (e.g. the Holm method [7]) to an *α* error. The program uses the method of Roldán-Nofuentes et al. [18], and as an individual method the Kosinski method is used (calculating the weighted generalized score statistic) since its performance is better than that of the rest of the methods.

Regarding the confidence intervals for the difference between the two *PPVs* and between the two *NPVs*, these are obtained inverting the statistic of the Kosinski method, i.e.

$$ {PPV}_1-{PPV}_2\in \hat{P}{PV}_1-\hat{P}{PV}_2\pm {z}_{1-\alpha /2}\sqrt{\left\{\hat{P}{PV}_p\left(1-\hat{P}{PV}_p\right)-2{C}_p^{PPV}\right\}\left(\frac{1}{n_{10}+{n}_{11}}+\frac{1}{n_{01}+{n}_{11}}\right)} $$

and

$$ {NPV}_1-{NPV}_2\in \hat{N}{PV}_1-\hat{N}{PV}_2\pm {z}_{1-\alpha /2}\sqrt{\left\{\hat{N}{PV}_p\left(1-\hat{N}{PV}_p\right)-2{C}_p^{NPV}\right\}\left(\frac{1}{n_{00}+{n}_{01}}+\frac{1}{n_{00}+{n}_{10}}\right)}. $$

### The “compbdt” program

The “compbdt” program is a program written with R software [3] which allows us to estimate and compare the previous parameters of two diagnostic test. The program is run with the command

$$ \mathrm{compbdt}\left({s}_{11},{s}_{10},{s}_{01},{s}_{00},{r}_{11},{r}_{10},{r}_{01},{r}_{00}\right) $$

when *α* = 5%, and with the command

$$ \mathrm{compbdt}\left({s}_{11},{s}_{10},{s}_{01},{s}_{00},{r}_{11},{r}_{10},{r}_{01},{r}_{00},\alpha \right) $$

when *α* ≠ 5%. Firstly, the program checks that the values introduced are viable (i.e., that there are no negative values, values of frequencies with decimals, etc.…) and that the estimated Youden index of each diagnostic test is greater than 0 (a necessary condition for every binary diagnostic test). The program also checks that it is possible to estimate and compare all of the parameters. If this is not possible (for example, when there are too many frequencies equal to 0), the program provides a message alerting to the error or the impossibility of estimating or comparing the parameters. By default, the program shows the numerical results with three decimal figures, a number which may be modified changing the command “decip <- 3” at the start of the code of the program.

Once it is established that it is possible to carry out the study, firstly the disease prevalence is estimated and we then estimate and compare the sensitivities and specificities, the likelihood ratios and the predictive values, following the methods described in the previous Section. For each type of parameter (*Se* and *Sp, PLR* and *NLR, PPV* and *NPV*), we calculate its estimation, standard error and confidence interval to 100(1 − *α*)%. Regarding the comparisons, if the global hypothesis test is significant, then the program solves the individual hypothesis tests along with the Holm method [7] (which is a less conservative method than the Bonferroni method) to a set *α* error. For the hypothesis tests which are declared significant, the confidence intervals are calculated for the difference (or ratio) of the parameters. These intervals are always calculated in such a way that they are positive (for the sensitivities, specificities and predictive values), and higher than 1 for the *LRs*, indicating the diagnostic test (*Test* 1 or *Test* 2) for which the parameter is estimated to be greater. If the global hypothesis test is not rejected, then the homogeneity of the parameters of both diagnostic tests is not rejected. In this situation, we do not calculate the confidence intervals for the difference or ratio of the parameters (since the homogeneity of the parameters is not rejected).

Furthermore, when the null hypothesis of the global hypothesis test is not rejected (and as long as the estimations are different), the program estimates the probability of making a type II error through Monte Carlo simulations. For this purpose, the program generates 10,000 random samples of a multinomial distribution with the same size as the original sample and as probabilities the relative frequencies observed in the original sample. The random samples are generated in such a way that in all of them it is possible to estimate the parameters and apply the hypothesis tests. Therefore, if for one generated sample it is not possible to apply a hypothesis test, then another sample is generated instead until completing the 10,000 samples. The estimation of the probability of making a type II error is based on the data observed in the original sample i.e. the probability of making a type II error is estimated assuming that subject to the alternative hypothesis the aim is to find a difference between the parameters such as the one observed in the original sample. The estimation of this probability is of great use for researchers as the non-rejection of the null hypothesis with a probability of making a type II error greater than 20% (a value which is normally considered to be a maximum value for this probability) indicates that the null hypothesis is not reliable, and it is necessary to increase the sample size. If in each global hypothesis test the alternative hypothesis test is accepted, then the program shows the estimated power of the test (one less the probability of making a type II error).

The results obtained comparing the sensitivities and specificities are recorded in the file “Results_Comparison_Accuracies.txt”, those obtained when comparing the *LRs* are recorded in the file “Results_Comparison_LRs.txt”, and those obtained when comparing the *PVs* are recorded in the file “Results_Comparison_PVs.txt”.

## Results

The “compbdt” program has been applied to the study of Weiner et al. [19] on the diagnosis of coronary artery disease, which is a classic example to illustrate statistical methods to compare parameters of two diagnostic tests. Weiner et al. [19] studied the diagnosis of coronary artery disease (*CAD*) using as diagnostic tests the exercise test (*Test* 1) and the clinical history of chest pain (*Test* 2), and the coronary angiography as the gold standard. Table 2 shows the frequencies obtained by applying three medical tests to a sample of 871 individuals.

**Table 2:** Study of Weiner et al

	Test 1 positive		Test 1 negative		
	Test 2 positive	Test 2 negative	Test 2 positive	Test 2 negative	Total
CAD	473	29	81	25	608
No CAD	22	46	44	151	263
Total	495	75	125	176	871

Running the “compbdt” program with the command

$$ \mathrm{compbdt}\left(473,29,81,25,22,46,44,151\right) $$

the following results are obtained:

### Prevalence of the disease

Estimated prevalence of the disease is 69.805% and its standard error is 0.016. 95% confidence interval for the prevalence of the disease is (66.681%; 72.768%).

### Comparison of the accuracies (sensitivities and specificities)

Estimated sensitivity of Test 1 is 82.566% and its standard error is 0.015. 95% confidence interval for the sensitivity of Test 1 is (79.363%; 85.389%).

Estimated sensitivity of Test 2 is 91.118% and its standard error is 0.012. 95% confidence interval for the sensitivity of Test 1 is (88.61%; 93.148%).

Estimated specificity of Test 1 is 74.144% and its standard error is 0.027. 95% confidence interval for the specificity of Test 1 is (68.557%; 79.087%).

Estimated specificity of Test 2 is 74.905% and its standard error is 0.027. 95% confidence interval for the specificity of Test 1 is (69.358%; 79.787%).

Wald test statistic for the global hypothesis test H0: (Se1 = Se2 and Sp1 = Sp2) is 25.662. Global *p*-value is 0.

Applying the global Wald test (to an alpha error of 5%), we reject the hypothesis H0: (Se1 = Se2 and Sp1 = Sp2). Estimated power (to an alpha error of 5%) is 99.8%.

Investigation of the causes of significance:

McNemar test statistic (with cc) for H0: Se1 = Se2 is 23.645 and the two-sided p-value is 0.

McNemar test statistic (with cc) for H0: Sp1 = Sp2 is 0.011 and the two-sided p-value is 0.991.

Applying the Holm method (to an alpha error of 5%), we reject the hypothesis H0: Se1 = Se2 and we do not reject the hypothesis H0: Sp1 = Sp2.

Sensitivity of Test 2 is significantly greater than sensitivity of Test 1. 95% confidence interval for the difference Se2 - Se1 is (5.192%; 11.857%).

### Comparison of the likelihood ratios

Estimated positive LR of Test 1 is 3.193 and its standard error is 0.339. 95% confidence interval for the positive LR of Test 1 is (2.61; 3.952).

Estimated positive LR of Test 2 is 3.631 and its standard error is 0.39. 95% confidence interval for the positive LR of Test 1 is (2.962; 4.505).

Estimated negative LR of Test 1 is 0.235 and its standard error is 0.022. 95% confidence interval for the negative LR of Test 1 is (0.195; 0.283).

Estimated negative LR of Test 2 is 0.119 and its standard error is 0.016. 95% confidence interval for the negative LR of Test 2 is (0.09; 0.153).

Test statistic for the global hypothesis test H0: (PLR1 = PLR2 and NLR1 = NLR2) is 23.438. Global p-value is 0. Applying the global hypothesis test (to an alpha error of 5%), we reject the hypothesis H0: (PLR1 = PLR2 and NLR1 = NLR2). Estimated power (to an alpha error of 5%) is 99.78%.

Investigation of the causes of significance:

Test statistic for H0: PLR1 = PLR2 is 0.898 and the two-sided p-value is 0.369.

Test statistic for H0: NLR1 = NLR2 is 4.663 and the two-sided p-value is 0.

Applying the Holm method (to an alpha error of 5%), we do not reject the hypothesis H0: PLR1 = PLR2 and we reject the hypothesis H0: NLR1 = NLR2. Negative likelihood ratio of Test 1 is significantly greater than negative likelihood ratio of Test 2. 95% confidence interval for the ratio NLR1 / NLR2 is (1.412; 2.554).

### Comparison of the predictive values

Estimated positive PV of Test 1 is 88.07% and its standard error is 0.014. 95% confidence interval for the positive PV of Test 1 is (85.17%; 90.498%).

Estimated positive PV of Test 2 is 89.355% and its standard error is 0.012. 95% confidence interval for the positive PV of Test 2 is (86.698%; 91.562%).

Estimated negative PV of Test 1 is 64.784% and its standard error is 0.028. 95% confidence interval for the negative PV of Test 1 is (59.246%; 69.976%).

Estimated negative PV of Test 2 is 78.486% and its standard error is 0.026. 95% confidence interval for the negative PV of Test 2 is (73.024%; 83.151%).

Wald test statistic for the global hypothesis test H0: (PPV1 = PPV2 and NPV1 = NPV2) is 25.944. Global p-value is 0.

Applying the global hypothesis test (to an alpha error of 5%), we reject the hypothesis H0: (PPV1 = PPV2 and NPV1 = NPV2). Estimated power (to an alpha error of 5%) is 99.26%.

Investigation of the causes of significance:

Weighted generalized score statistic for H0: PPV1 = PPV2 is 0.807 and the two-sided p-value is 0.369.

Weighted generalized score statistic for H0: NPV1 = NPV2 is 22.502 and the two-sided p-value is 0.

Applying the Holm method (to an alpha error of 5%), we do not reject the hypothesis H0: PPV1 = PPV2 and we reject the hypothesis H0: NPV1 = NPV2.

Negative PV of Test 2 is significantly greater than negative PV of Test 1. 95% confidence interval for the difference NPV2 - NPV1 is (8.041%; 19.363%).

These outputs obtained when running the program allow researchers to interpret the results easily. First, for each type of parameters, all parameters are estimated and the corresponding global test is solved. In summary, the three global hypothesis tests are rejected and then the causes of the significance of each global test are investigated. For individual hypothesis tests that are declared significant, it is indicated which is the diagnostic test for which the parameter is greater, calculating the corresponding confidence interval. Due to the high sample size, the estimated power for each of the global tests is very high (close to 100%).

In R, an alternative program to “compbdt” is the DTComPair package [20]. The DTComPair package estimates the same parameters as the “compbdt” and compares the parameters individually, i.e. solving each hypothesis test to an *α* error. Table 3 shows the results obtained when applying the DTComPair package with *α* = 5% (the estimations of the parameters and their standard errors are not shown as they are the same as those obtained with the “compbdt” program). The conclusions obtained are similar to those obtained with the “compbdt” program, although this program uses methods with better asymptotic behaviour.

**Table 3:** Results obtained with the DTComPair package

Confidence intervals for the parameters of each diagnostic test (95% confidence)		
	*Test* 1	*Test* 2
Sensitivity	79.550%; 85.582%	88.857%; 93.380%
Specificity	68.853%; 79.436%	69.665%; 80.145%
Positive LR	2.594; 3.931	2.942; 4.481
Negative LR	0.195; 0.284	0.091; 0.154
Positive PV	85.409%; 90.731%	86.927%; 91.783%
Negative PV	59.388%; 70.180%	73.403%; 83.570%
Comparison of the parameters of the two diagnostic tests (*α* = 5%)		
Sensitivities		
McNemar test statisics: test statistic = 24.582, p ‐ value = 0		
Exact test: p ‐ value = 0		
95% Tango confidence interval for *Se*_2_ − *Se*_1_: 5.278%; 11.966		
Specificities		
McNemar test: test statistic = 0.044, p ‐ value = 0.833		
Exact test: two ‐ sided p ‐ value = 0.916		
Likelihood ratios (Method of Leisenring et al. [21] and Pepe [1])		
Positive *LRs*: test statistic = − 0.898, p ‐ value = 0.369		
Negative *LRs*: test statistic = 4.663, p ‐ value = 0 95% confidence interval for *NLR*_1_/*NLR*_2_: 1.487; 2.644		
Predictive values (Method of Leisenring et al. [13])		
Positive *PVs*: test statistic = 0.802, p ‐ value = 0.371		
Negative *PVs*: test statistic = 23.579, p ‐ value = 0		
Predictive values (Method of Kosinski [14])		
Positive *PVs*: test statistic = 0.807, p ‐ value = 0.369		
Negative *PVs*: test statistic = 22.502, p ‐ value = 0		
Relative predictive values (Method of Moskowitz and Pepe [22])		
Positive *PVs*: test statistic = − 0.895, p ‐ value = 0.371		
Negative *PVs*: test statistic = − 4.737, p ‐ value = 0 95% confidence interval for *NPV*_1_/*NPV*_2_: 0.762; 0.894		

## Conclusions

The comparison of the performance of two diagnostic tests subject to a paired design is an important topic in Medicine. Many studies have been carried out on statistical methods to estimate and compare parameters of two binary diagnostic tests subject to this type of design. In the “compbdt” program the most efficient methods have been implemented, in terms of coverage and width for the confidence intervals and in terms of type I error and power for the hypothesis tests, developed up to the present day. The comparisons of the three types of parameters (sensitivities and specificities, likelihood ratios and predictive values) are based on solving the global hypothesis tests. For each type of parameter, the program solves the global test and if this is not significant to an *α* error then we do not reject the homogeneity of the parameters of both diagnostic tests; if the global test is significant to an *α* error then the causes of the significance are investigated solving the individual hypothesis tests along with Holm’s method of multiple comparison to an *α* error. This procedure is very similar to analysis of variance. If for each type of parameter we directly solve each one of the individual hypothesis tests to an *α* error, it is possible to obtain mistaken results. Two examples of this are explained in the articles by Roldán-Nofuentes and Sidaty-Regad [6] and Roldán-Nofuentes et al. [18].

The program requires installing the *R* software, which is freely available at the URL “https://www.r-project.org”, and it is necessary for the data observed to have the structure given in Table 1. The program provides all of the results necessary so that the researcher can make interpretations in a simple way. Another contribution made by this program is the estimation of the probability of making a type II error based on the data observed in the sample through Monte Carlo simulations, data which provides information about the reliability of the null hypothesis when the hypothesis test is not significant. The program has been applied to a classic example of this topic. On an Intel Core i7 3.40 GHz computer the program has been run in around 7 s.

With respect to the DTComPair package [20], the “compbdt” program uses methods with better asymptotic behaviour and has the following advantages:

- For a binomial proportion (such as the sensitivity, specificity and predictive values of each diagnostic test), the DTComPair package uses the Agresti and Coull interval [23]. The “compbdt” uses the interval of Yu et al. [4], which has a better coverage than that of Agresti and Coull.
- The DTComPair uses the interval of Simel et al. [24] for the positive (negative) likelihood ratio of each diagnostic test, an interval which, as is well known, does not have a good coverage when the samples are not very large. The “compbdt” program uses the interval of Martín-Andrés and Álvarez-Hernández, which is the interval with the best coverage for the ratio of two independent binomial proportions (such as the positive and negative likelihood ratios).
- The DTComPair package compares the parameters individually, which can lead to mistakes [6, 18]. The “compbdt” program is based on the simultaneous comparisons of the parameters and on research into the causes of the significance when the global tests are significant.
- The DTComPair package calculates three confidence intervals for the difference of the two sensitivities (specificities): Wald (with or without *cc*), Agresti and Min [25], and Tango [26]. Fagerland et al. [8] have shown that the Wald interval with Bonett-Laplace adjustment (interval implemented in the “compbdt” program) has an asymptotic behaviour very similar to that of Tango, and that both intervals have a better behaviour than that of Agresti and Min. The advantage of the Wald interval with Bonett-Laplace adjustment is that this interval has closed-form expression.
- The DTComPair package calculates confidence intervals for the ratio of *LRs* based on regression models [1, 21]. The “compbdt” program uses confidence intervals with better asymptotic behaviour [12].
- The “compbdt” program estimates the power or probability of making a type II error, depending on whether or not the alternative hypothesis is accepted or not the null hypothesis is rejected, based on the data observed in the sample through Monte Carlo simulations.
- The DTComPair package only provides numerical results, whereas the “compbdt” program also interprets them, which is of great use for the clinician.

The application of the “compbdt” program requires the results of both diagnostic tests and the gold standard to be known for all of the individuals in the sample. If the result of a diagnostic test is unknown for any individual, and this missing data is random due to chance (the missing data mechanism is missing at random), this data can always be imputed applying some method of imputation and then it is possible to use the program to solve the problem of comparison of the parameters. The program also requires knowledge of the discordant frequencies (*s*_*ij*_ and *r*_*ij*_ with *i* ≠ *j*), since these are necessary to be able to solve the hypothesis tests. If the researcher wants to use the “compbdt” program to repeat the results of a study and we do not know the discordant frequencies but we do know an estimation of the Cohen kappa coefficient (or another measure of association) between the diagnostic tests in diseased individuals and in non-diseased individuals, then it is possible to use both estimations to obtain the values of the discordant frequencies. The “compbdt” program is available as supplementary material of this manuscript.

Finally, the “compbdt” program can also be applied when the sampling is case-control, i.e. the two diagnostic tests are applied to two samples, one of *n*_1_ diseased individuals and another one of *n*_2_ non-diseased individuals. In this situation, the frequencies *s*_*ij*_ correspond to the case sample (with ${n}_1=\sum \limits_{i,j=0}^1{s}_{ij}$) and the frequencies *r*_*ij*_ correspond to the control sample (with ${n}_2=\sum \limits_{i,j=0}^1{r}_{ij}$). Subject to this sampling, it is necessary to take into account the fact that the results obtained for the prevalence and all of the results obtained for the predictive values are not valid, since from a case-control sample it is not possible to obtain an estimation of the disease prevalence (the value *n*_1_/(*n*_1_ + *n*_2_) is not an estimation of the prevalence since the sample sizes *n*_1_ and *n*_2_ are set by the researcher).

## Availability and requirements

Project name: Comparison of binary diagnostic tests.

Project home page: https://www.ugr.es/~bioest/

Operating system(s): Platform independent.

Programming language: R.

Other requirements: R 3.6.1 or above.

License: GPL-2.

Any restrictions to use by non-academics: none.

## Supplementary information

**Additional file 1.**

## Data Availability

All data generated or analyzed during the current study are included in this published article. The program “compbdt” is available as supplementary material of this manuscript.

## Abbreviations

CAD: Coronary artery disease
LR: Likelihood ratio
p: Prevalence of the disease
PLR: Positive likelihood ratio
PV: Predictive value
PPV: Positive predictive value
NLR: Negative likelihood ratio
NPV: Negative predictive value
Se: Sensitivity
Sp: Specificity
